# Accurate prediction of terahertz spectra of molecular crystals of fentanyl and its analogs

**DOI:** 10.1038/s41598-021-83536-y

**Published:** 2021-02-18

**Authors:** Chun-Hung Wang, Anthony C. Terracciano, Artёm E. Masunov, Mengyu Xu, Subith S. Vasu

**Affiliations:** 1 NanoScience Technology Center, University of Central Florida, 12424 Research Parkway, Orlando, FL 32826 USA; 2 Department of Mechanical and Aerospace Engineering, University of Central Florida, 4000 Central Florida Blvd, Orlando, FL 32816 USA; 3 Center for Advanced Turbomachinery and Energy Research, University of Central Florida, 4000 Central Florida Blvd, Orlando, FL 32816 USA; 4 South Ural State University, Lenin Pr. 76, Chelyabinsk, Russia 454080; 5 National Research Nuclear University MEPhI, Kashirskoye Shosse 31, Moscow, Russia 115409; 6 Department of Statistics and Data Science, University of Central Florida, 4000 Central Florida Blvd, Orlando, FL 32816 USA

**Keywords:** Chemistry, Materials science, Physics

## Abstract

Fentanyl is a potent synthetic opioid pain reliever with a high bioavailability that can be used as prescription anesthetic. Rapid identification via non-contact methods of both known and emerging opioid substances in the fentanyl family help identify the substances and enable rapid medical attention. We apply PBEh-3c method to identify vibrational normal modes from 0.01 to 3 THz in solid fentanyl and its selected analogs. The molecular structure of each fentanyl analog and unique arrangement of H-bonds and dispersion interactions significantly change crystal packing and is subsequently reflected in the THz spectrum. Further, the study of THz spectra of a series of stereoisomers shows that small changes in molecular structure results in distinct crystal packing and significantly alters THz spectra as well. We discuss spectral features of synthetic opioids with higher potency than conventional fentanyl such as ohmefentanyl and sufentanil and discover the pattern of THz spectra of fentanyl analogs.

## Introduction

Fentanyl (*N*-phenyl-*N*-[1-(2-phenylethyl)piperidin-4-yl]propionanilide)^1^ is one kind of synthetic opioid used for pain relief, which has high affinity for μ-opioid receptor and acts as an agonist with higher potency than morphine. Its effective dose (ED_95_) is 0.45–0.60 μg/kg, partly due to its 92% bioavailability^2,3^. A comprehensive review of the history of fentanyl and its analogs has been published in 2018 by Armenian et al.^4^ Carfentanil^5^, sufentanil^6^, alfentanil^7^, and remifentanil^8^ are fentanyl analogs with additional functional groups which enable a higher potency than fentanyl itself.

Once in the body, fentanyl metabolism begins with N-oxidative dealkylation and the products are eventually excreted with the urine^9,10^. Fentanyl can be administered via inhalation, ingestion, oral exposure, injection, or via transdermal means^11,12^. It is primarily produced in the form of powdered substances, typically white. When obtained outside of a pharmaceutical setting, it may be mixed with heroin and cutting agents or pressed into counterfeit opioid prescription pills^13^. Fentanyl may even be weaponized in the form of aerosol^14^. Widespread use of fentanyl as either a pure compound or mixture with other drugs poses a significant threat for first responders given its ample means of exposure and effective dosing^15^.

Several existing methods may be used for fentanyl and other narcotic detection including: ion mobility spectrometry^16,17^, electron impact mass spectrometry^18^, microtiter plate enzyme analysis^19^, radio immunoassays^20^, thermal desorption direct analysis in real-time mass spectrometry^21^, surface enhanced Raman spectroscopy^22,23^, and differential pulse voltammetry^24^. However, these methods require handling of the substance and/or taking samples of the bodily fluids. Fentanyl analogs have similar structure, but exhibit distinct functional groups, which complicates detection of such synthetic opioids. Additionally, clandestine laboratories do not have stringent protocols for ensuring the proper reaction occurs and novel variations. Thus, desirable means of fentanyl detection must possess the following features: (i) non-contact identification of fentanyl and its derivatives; (ii) accurate detection with minimal false positives and false negatives; (iii) be simple for an operator with no scientific background; and (iv) provide results in seconds.

The method for fentanyl identification which satisfies the above criteria is THz spectroscopy which employs electromagnetic fields in the THz region (0.1–3.0 THz) and is applicable to imaging and sensing^25,26^. Non-polar dielectric objects such as clothes, plastics, paper envelope, and luggage are semi-transparent to THz radiation minimizing obscuring effects of containers^27,28^. In addition, the narrow range 0.1–3.0 THz of low vibrational frequency between far infrared (IR) and microwave may provide spectroscopic fingerprint, capable to detect fentanyl analogs accurately.

To facilitate THz identification of fentanyl, it is advantageous to assemble a database of fentanyl THz spectra. This can be done, for instance, by quantum mechanical (QM) density-functional theory (DFT) calculations in solid state. Such efforts will enable chemometrics techniques for understanding and validation of reflectance spectroscopy experiment, and the fast prediction techniques by machine learning (ML) methods. In this work, we will establish a library of THz spectra of fentanyl and its analogs via QM calculation.

**Figure 1:**
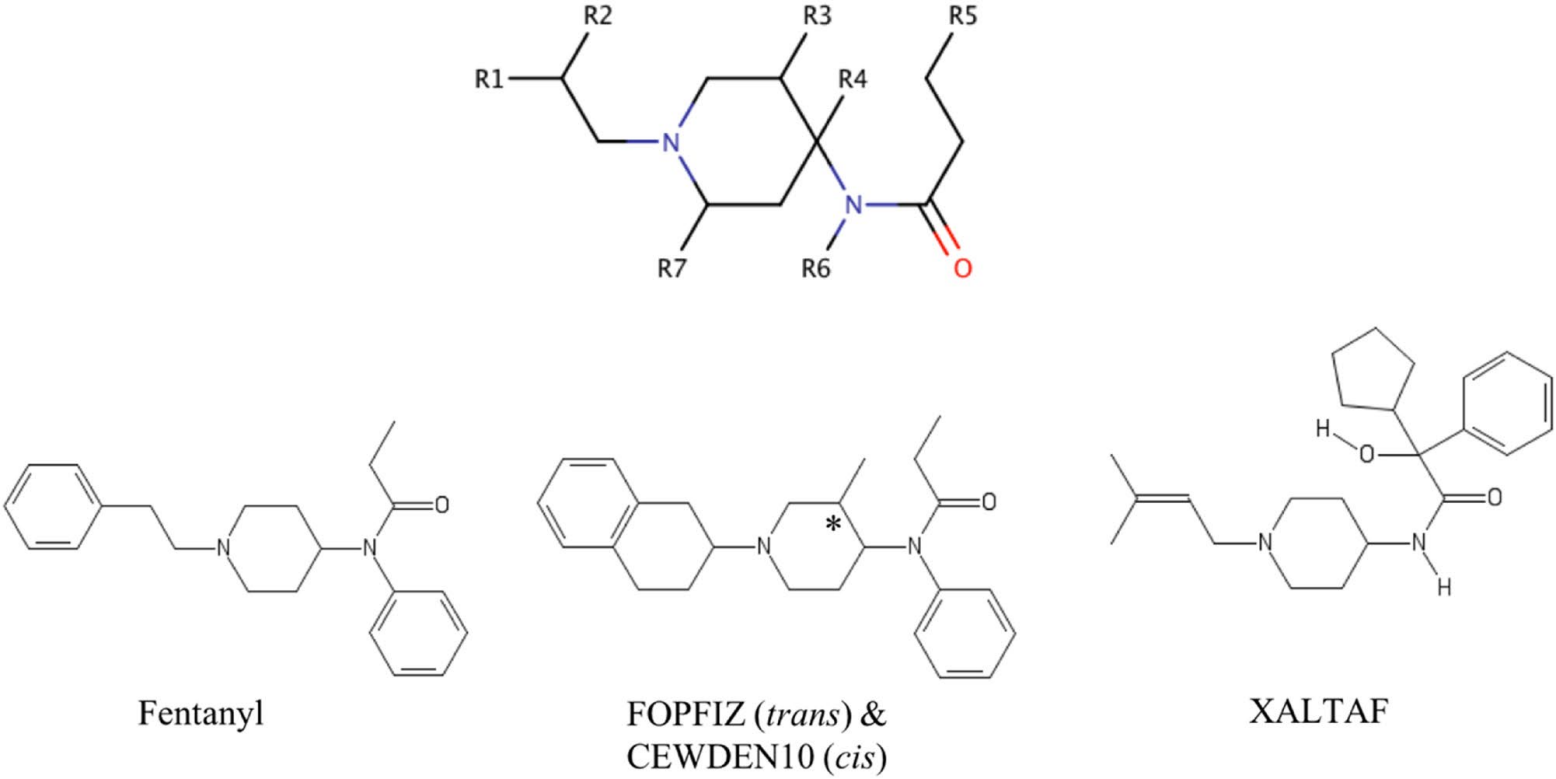
Structure of fentanyl, selected analogs with stereoisomer FOPFIZ/CEWDEN10, and XALTAF; “*” indicates chiral center.

Molecule of fentanyl includes three functional moieties: *N*-phenyl-propanamide, piperidine ring, and *N*-phenylethyl ring. Fentanyl analogs we selected for spectral predictions are those with altered pharmacology and toxicology (shown on Fig. 1 and Table 1). Substitution of one methyl group into the piperidine ring in fentanyl results in 3-methylfentanyl^29^. Substitution of a hydroxyl group between piperidine and phenyl group in 3-methylfentanyl leads to ohmefentanyl^30,31^, which contains the multiple chiral centers denoted by the “*” at particular atom(s). Adding other functional groups to the structure of fentanyl can produce BIYTAF^32^ (*N*-(1-(2-hydroxy-2-phenylethyl)-4-piperidyl)-*N*-phenylpropanamide), TACDOS/TACDUY^33^ (5-oxo-5-(phenyl(1-(2-phenylethyl)piperidinium-4-yl)amino)pentanoate, JINPAX (*2R*,*5S*)^34^ & VEYCIL (*2S*,*5S*)^35^ (2,5-Dimethyl-1-(2-phenylethyl)-4-(*N*-propionylanilino)piperdine), FOPFIZ/CEWDEN10^36^ (*trans*/*cis*-*N*-(3-Methyl-1-(2-(1,2,3,4-tetrahydro)naphthyl)-piperidin-4-yl)-*N*-phenylpropanamide), FBPIPA^37^ (*N*-(1-(3-(*p*-fluorobenzoyl)propyl)-4-piperidyl)propionanilide), BEGZUI^38^ (*N*-(1-(2-Phenylethyl)-4-piperidyl)-*N*-(1-phenyl-3-pyrazolyl)propanamide), IFIGIN^39^ ((*1R*)-2-[(*3R*,*4S*)-3-Methyl-4-(*N*-phenyl-*N*-propionylamino)piperidin-1-yl]-1-phenylethyl *p*-Bromobenzoate), ZIZPON^40^ (1-(2-Phenylethyl)-4-((3-trimethylgermylpropanoyl)phenylamino)piperidinium), or XALTAF^41^ ((2R)-*N*-(1-(4-Methyl-3-pentenyl)piperidin-4-yl)-2-cyclopentyl-2-hydroxy-2-phenylacetamide).

**Table 1:** Molecular structures of fentanyl and its analogs.

	R1	R2	R3	R4	R5	R6	R7
Fentanyl	Ph	H	H	H	H	Ph	H
Ohmefentanyl	Ph	OH	Me	H	H	Ph	H
3-Methylfentanyl	Ph	H	Me	H	H	Ph	H
BIYTAF	Ph	OH	H	H	H	Ph	H
TACDOS/TACDUY	Ph	H	H	H	CH_2_CO_2_H	Ph	H
FBPIPA	*p*-FPhC(O)CH_2_	H	H	H	H	Ph	H
BEGZUJ	Ph	H	H	H	H	1-phenyl-3-pyrazolyl	H
JINPAX/VEYCIL	Ph	H	Me	H	H	Ph	Me
IFIGIN	Ph	*p*-BrPhCO_2_	Me	H	H	Ph	H
ZIZPON	Ph	H	H	H	GeMe_3_	Ph	H
Carfentanil	Ph	H	H	CO_2_Me	H	Ph	H
R-30490	Ph	H	H	CH_2_OMe	H	Ph	H
Sufentanil	2-thiophenyl	H	H	CH_2_OMe	H	Ph	H
Alfentanil	4-ethyl-5-oxo-tetrazol-1-yl	H	H	CH_2_OMe	H	Ph	H
Thiofentanil	2-thiophenyl	H	H	CO_2_Me	H	Ph	H

The presence of the one (FOPFIZ/CEWDEN10), two (JINPAX/VEYCIL), and three (ohmefentanyl) chiral centers results in distinct packing features for crystallized stereoisomers. While it has not been considered here, we also expect hydrates to decrease the intensity of observed absorption peaks as the phonon modes transmitted within the lattice will be damped as water is included^42^. In order to compare with experimental results, 4-methoxymethylfentanyl (R-30490^43^, *N*-(4-(Methoxymethyl)-1-(2-phenylethyl)-4-piperidinyl)-*N*-phenyl-propanamide), sufentanil^6^, alfentanil^7^, and thiofentanil^44^ are studied computationally as well (see Table 1). The stereoisomers have distinct crystal packing and thermodynamic parameters, which determine the difference in properties like solubility and bioavailability.

The detection of fentanyl by THz spectra is possible due to unique phonon properties (molecular motion). Each phonon mode in the given wavelength range corresponds to a hindered molecular rotation (libration), coupled to intramolecular vibrations. Differences in the molecular structure of the fentanyl analogs leads to distinctly different crystalline packing and hence THz spectra. In general, long-distance intermolecular interaction determines the strength and the position of peaks on THz spectra in the solid state. Specific intermolecular interactions such as hydrogen bonding and π-π stacking tend to have significant influence of THz frequency shift^45^.

In order to accurately describe long-distance dispersion interactions for organic molecular solids, we have decided to apply Grimme’s PBEh-3c method^46^, based on corrected Perdew-Burke-Ernzerhoff (PBE)^47^ hybrid generalized-gradient-approximation functional. Conformational analyses of fentanyl and its analogs ohmefentanyl^48–50^, 3-methylfentanyl^49^, BIYTAF^48^, sufentanil^48^, and alfentanil^48,51^ have been studied previously in different groups by molecular dynamics and molecular docking to understand the docking site of fentanyl analogs to μ-opioid receptor and by DFT calculations to understand stable conformations in gas and aqueous phase. To the best of our knowledge, this is the first time THz spectra of fentanyl and its analogs in solid state had been predicted by DFT calculation. Other factors which may affect measured THz spectra (such as mixture of different fentanyl analogs and hydrates) can be aggressed in our future work by ML methods.

## Computational methods

All the crystal structures of the various fentanyl molecules were obtained from the Cambridge Structural Database^52^. The structures were preprocessed using cif2cell^53^ to convert crystallographic information file to the proper input file format. Some of the fentanyl analogs were missing hydrogen atoms, which were added geometrically using Molden program^54^. Marvin^55^ was used for drawing, displaying, and characterizing two-dimensional chemical structures and Mercury^56^ was used for visualizing three-dimensional geometry. Post-processing of the output files to produce the graphical representation of the absorption spectra and vibrational normal modes was done using CRYSPLOT^57^. Microsoft Excel 2019 was used for presenting absorption spectra^58^. CRYSTAL17^59^, was utilized for solid-state DFT calculations with periodic boundary conditions. Sufficiently large supercell (between 53 and 134 atoms) was constructed to ensure long range vibrations were accounted for. The multiplicity of the unit cell was determined by the ratio of the volume of the simulation supercell to the volume of a primitive cell. Corrections for basis-set superposition error and long-range dispersion interactions were included within PBEh-3c method^46^. This method is based upon the modified global hybrid functional PBEh, where percentage of Hartree–Fock exchange (42%) is adjusted to get correct average bond length. Modified double-ζ Gaussian basis set dubbed def2-mSVP^46,60^ is defined within the method as well. The benchmark study, validating the accuracy of the chosen computational method is described in detail in Supporting Information.

Full geometry optimization, including both atom coordinates and unit cell parameters (a, b, c, α, β, and γ), was applied to fully relax the geometry within the constraints of the space group symmetry. Threshold of convergence for crystal structure, based on the energy change between consecutive geometry optimization steps was set to 10^−8^ Bohr. Vibrational normal modes^61^ and IR intensities were calculated using the Berry phase method^62^. For the calculated vibrational frequencies, the scaling factor of 0.95 was applied for all of the spectral predictions^46^. The damping factor for the calculation of the Lorentzian linewidth at half maximum of absorption peak was set to 2.0 cm^−1^, in order to match the spectral shapes recorded at room temperature. Threshold on Self Consistent Field energy convergence was 10^−10^ Hartree. Truncation criteria for bielectronic integrals were as follows: overlap threshold for Coulomb integrals was 10^−7^, penetration threshold for Coulomb integrals was 10^−7^, overlap threshold for HF exchange integrals was 10^−7^, pseudo-overlap (HF exchange series) were 10^−7^ and 10^−14^. These criteria are based on well established guidelines which were described in Ref^63^.

## Results and discussion

### Geometry optimization, hydrogen bond, and charge distribution

Predicted unit cell parameters of fentanyl analogs are reported in Table S4. Their deviations from experimental values (collected in Table S3) are all within 0.3–0.9 Å and 1.2–5.0°. The changes of unit cell volume upon optimization was less than 5% with the only exception of FOPFIZ, where this change was ~ 10%.

The presence of O and N atoms in molecular structures of fentanyl and its analogs open the possibilities for the formation of hydrogen bonds (H-bonds). In fentanyl, TACDOS, and XALTAF, H-bond is formed by N atom of piperidine ring (N…H distances are 2.1, 1.6, and 2.1 Å, respectively). However, O and N atoms in *N*-phenyl-propanamide do not form H-bonds. Counterions in alfentanil and thiofentanil form Cl^−^…H interactions (~ 2.1 Å). Other sources of H-bond interaction include solvent molecules water, oxalic acid, or hydrogen sulfate.

Hirshfeld population analysis^64^ describes molecular charge density in terms of atomic contribution and allows one to examine the nature of the charge transfer between various species in each crystal structure. The magnitude of Hirshfeld atomic charges is less dependent by basis set and is typically smaller than that of Mulliken charges^65^. Of all the fentanyl and its analogs, atomic charge of O atom in *N*-phenyl-propanamide is − 0.555 ± 0.016, N atom in *N*-phenyl-propanamide is − 0.363 ± 0.047, and N atom in piperidine ring is − 0.189 ± 0.027 (neutral) or 0.031 ± 0.059 (if protonated, H atom is 0.248 ± 0.067). Hence, the charge distribution in fentanyl and its analogs is consistent.

### THz spectra of fentanyl and its analogs

**Figure 2:**
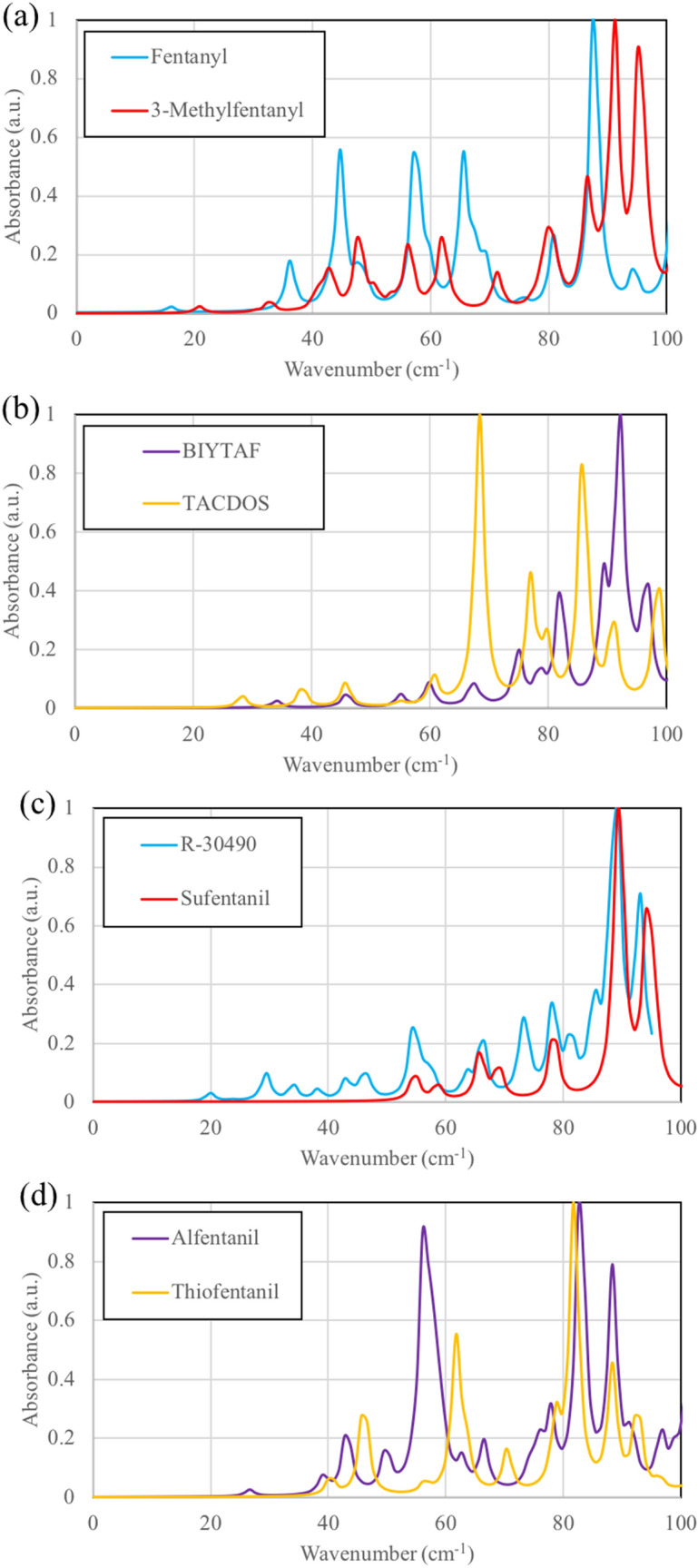
THz spectra of (**a**) fentanyl and 3-methylfentanyl, (**b**) BIYTAF and TACDOS, (**c**) R-30490 and sufentanil, and (**d**) alfentanil and thiofentanil.

Predicted THz absorption spectra of fentanyl and selected analogs are shown in Fig. 2. These correspond to polycrystalline samples (anisotropy is averaged out). Even one functional group difference results in a change of the crystal packing and thus the shape of absorption spectra. As one can see in Fig. 2, an additional methyl group for 3-methylfentanyl, a hydroxyl group for BIYTAF, and a carboxyl group for TACDOS results in different THz spectra. There are various medium peaks between 35 and 85 cm^−1^ of 3-methylfentanyl (Fig. 2a). Fentanyl, 3-methylfentanyl, and BIYTAF all have strongest peak above 85 cm^−1^. Normal mode visualization indicates that two peaks for fentanyl at 45 and 58 cm^−1^ arise from the intermolecular wiggling of T-shaped packing of the phenyl rings, which is not found in the other 3 analogs (Fig. 2a,b). The addition of methoxymethyl group on piperidine ring results in the appearance of several weak peaks in THz spectra of R-30490 (Fig. 2c) but no significant peaks like 45, 58, and 68 cm^−1^ in fentanyl and 68, 77, and 86 cm^−1^ in TACDOS (see Fig. 2a,b). Strong peaks arise from N…H H-bonds to piperidine ring in fentanyl and TACDOS. Comparison between TACDOS and TACDUY (with solvents water and methanol) is shown in Fig. S2. Medium to strong peaks between 25 and 75 cm^−1^ are from H-bonds between solvents water and methanol. For alfentanil and thiofentanil (Fig. 2d), the strong peaks at 56 and 62 cm^−1^ arise from Cl…H stretch between counterion and protonated piperidine ring. R4 group in both R-30490 and sufentanil (see Table 1) does not form strong H-bonds, and dispersion interactions produce small but sharp peaks below 80 cm^−1^.

**Figure 3:**
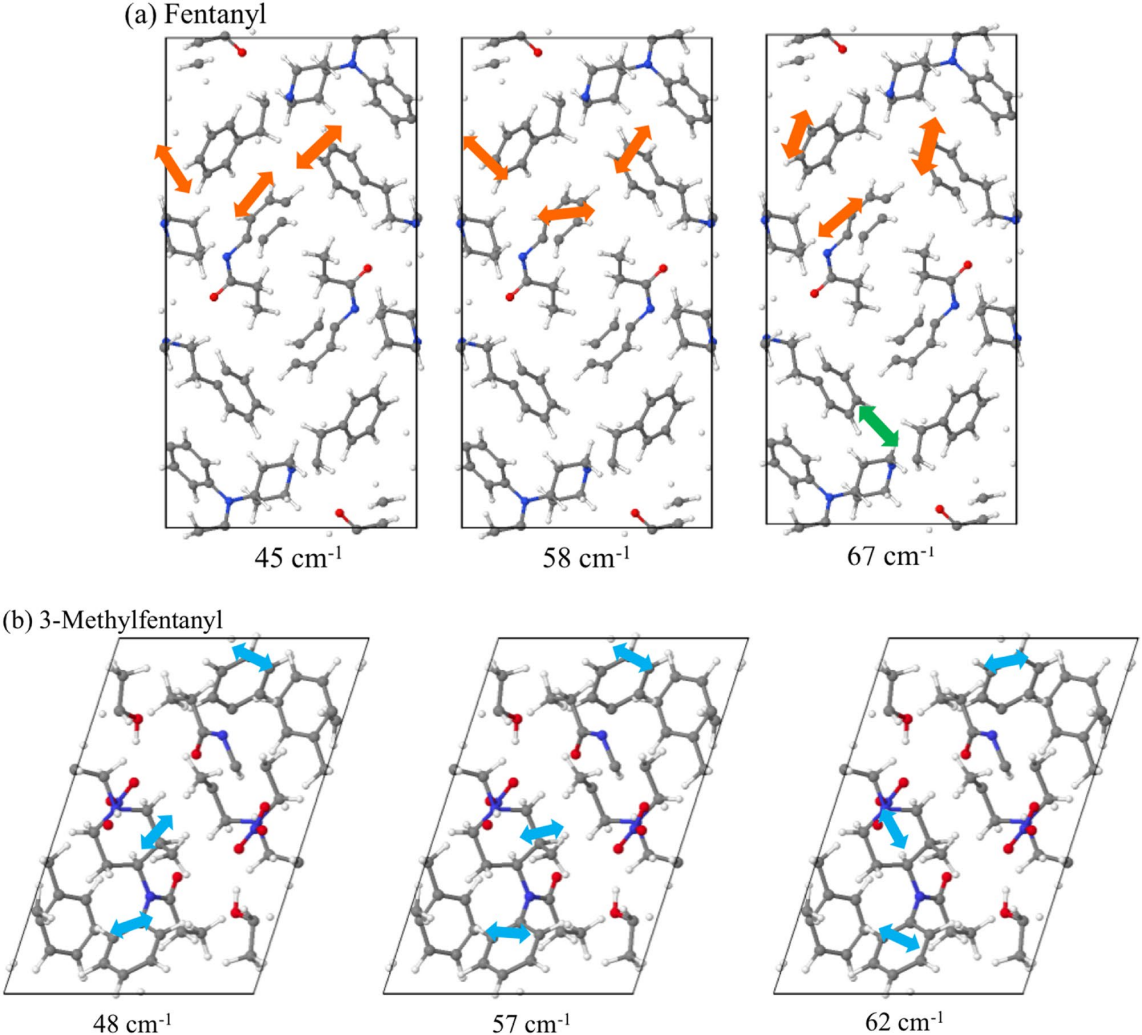
Normal modes of fentanyl in (**a**) 45, 58, and 67 cm^−1^ along with a-axis. Orange arrows show the vibrational direction of benzene and piperidine. Green arrow shows the formation of N…H H-bond. Normal modes of 3-methylfentanyl in (**b**) 48, 57, and 62 cm^−1^ along with b-axis. Blue arrows show the vibrational direction of benzene and 3-methylpiperidine. Carbon, hydrogen, nitrogen, and oxygen atoms are depicted as grey, white, blue, and red spheres, respectively.

Figure 3a shows the vibrational normal modes of fentanyl at 45, 58, and 67 cm^−1^ and of 3-methylfentanyl at 48, 57, and 62 cm^−1^ are visualized in Fig. 3b. The difference in one methyl group leads to a distinct crystal packing and normal modes of fentanyl and 3-methylfentanyl. Similarly, sufentanil where the phenyl ring is substituted by thiophene ring results in weak peaks near 57, 67, and 78 cm^−1^, while alfentanil with the substitution to tetrazole ring leads to strong peaks at 56, 85, and 88 cm^−1^.

### THz spectra of fentanyl analogs with chiral centers

**Figure 4:**
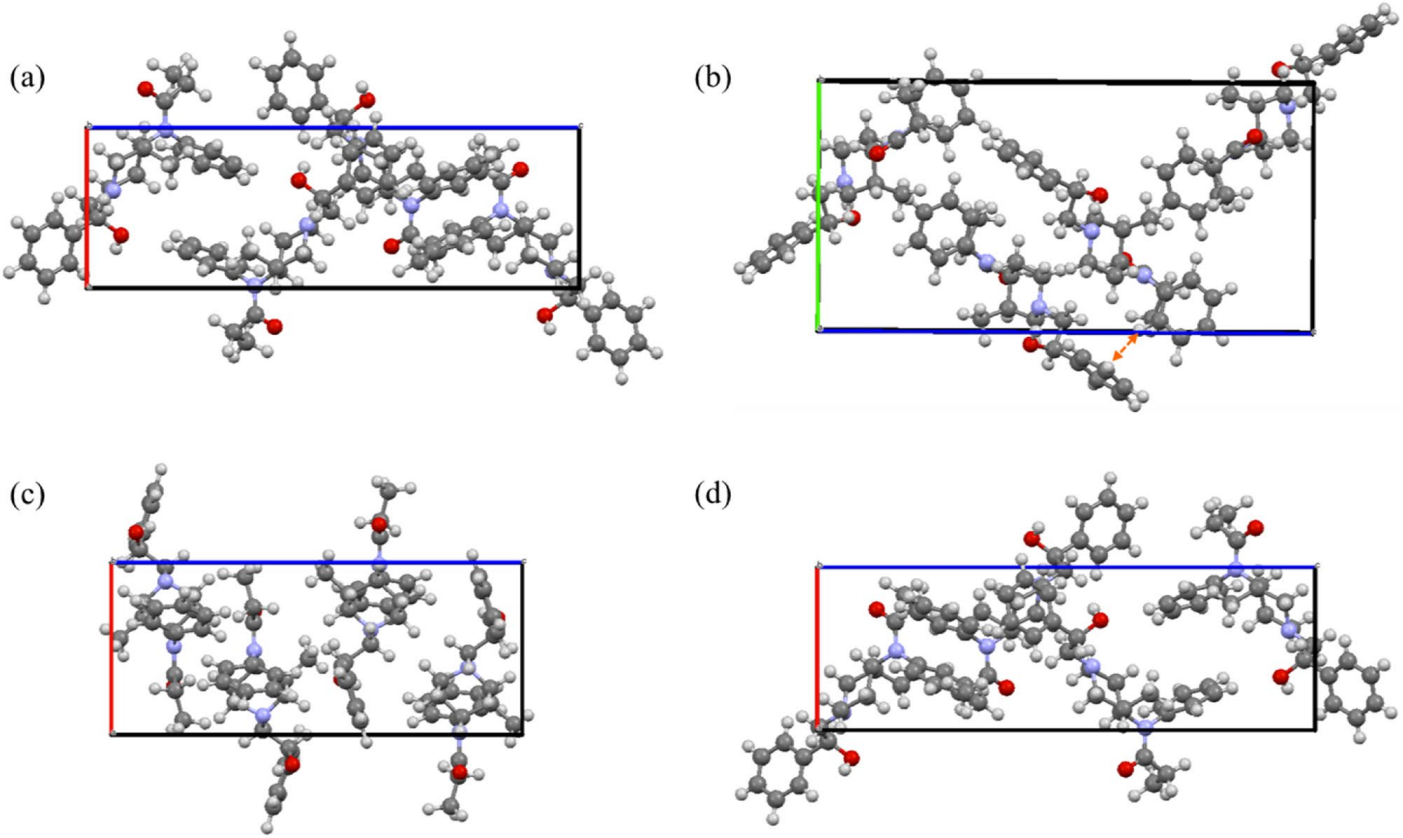
Crystal packing of ohmefentanyl stereoisomers (**a**) *cis*-(*2R*,*3R*,*4S*) viewed along with b-axis, (**b**) *cis*-(*2R*,*3R*,*4S*) along with a-axis, (**c**) *trans*-(*2S*,*3R*,*4R*) along with b-axis, and (**d**) *cis*-(*2S*,*3R*,*4R*) viewed along with b-axis. Carbon, hydrogen, nitrogen, and oxygen atoms are depicted as grey, white, light purple, and red spheres, respectively.

The mode of molecular packing influences THz spectra significantly. Even for fentanyl analogs of the same atomic composition, stereoisomers induce changes in the packing and location of librational centers. Figure 4 shows the example of different crystal packing formed by the 3 ohmefentanyl isomers from R2, R3, and R4 groups (see Table 1) along with *b*- or *a*-axis. Herringbone packing is visible when viewing *cis*-(*2R*,*3R*,*4S*) and *cis*-(*2S*,*3R*,*4R*) from 3 different directions in Fig. 4a,b,d. Orange arrow in Fig. 4b shows T-shaped interaction. However, parallel packing is seen when viewing *trans*-(*2S*,*3R*,*4R*) along a- and b-axes while herringbone packing is seen along c-axis (Fig. 4c). The orientation of OH in R2 and CH_3_ in R3 groups influences the packing significantly.

**Figure 5:**
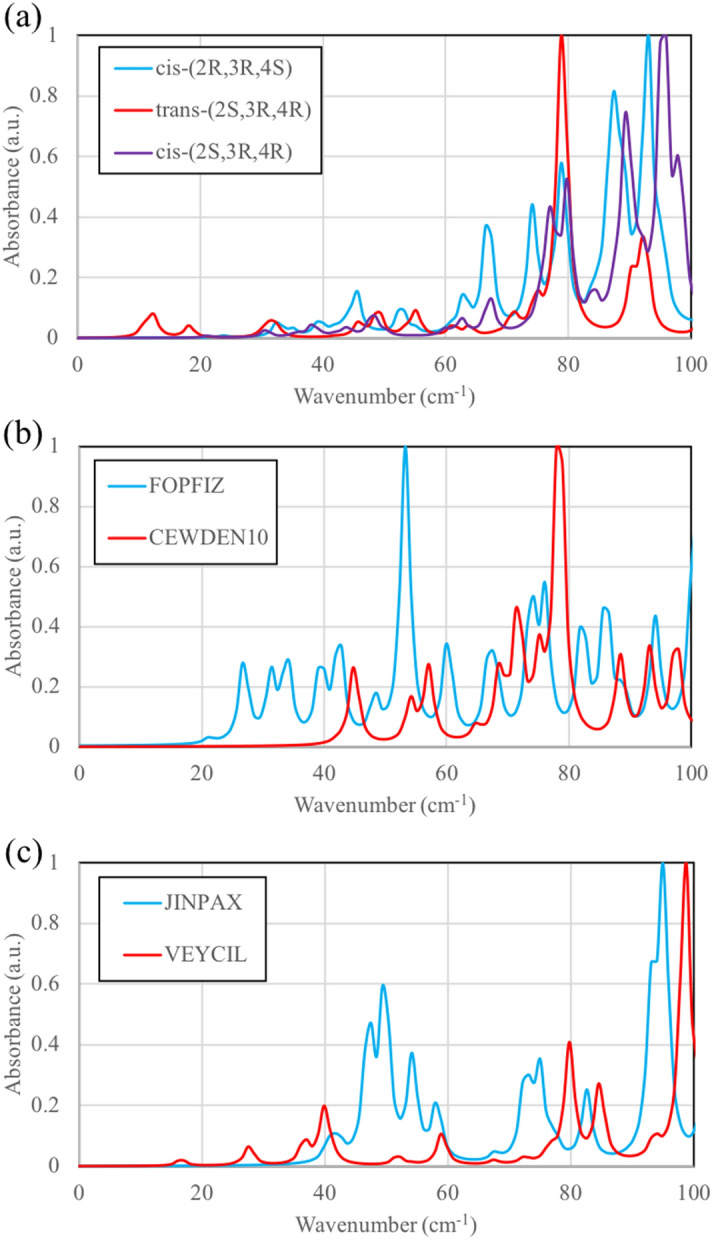
THz spectra of stereoisomers (**a**) ohmefentanyl (*cis*-(*2R*,*3R*,*4S*), *trans*-(*2S*,*3R*,*4S*), and *cis*-(*2S*,*3R*,*4R*)), (**b**) FOPFIZ (*trans*) and CEWDEN10 (*cis*), and (**c**) JINPAX (*2R*,*5S*) and VEYCIL (*2S*,*5S*).

THz absorption spectra of stereoisomers for selected fentanyl analogs are shown in Fig. 5. In ohmefentanyl (Fig. 5a) with stereoisomeric variations *cis*-(*2R*,*3R*,*4S*) and *cis*-(*2S*,*3R*,*4R*), similar THz spectra shows both the crystal packing include T-shaped interaction (Fig. 4a,d). There is merely a slight wavelength shift and it represents the packing dominates THz spectra. Intermolecular H-bond between ohmefentanyl molecules is absent even in the presence of OH group in R2 (see Table 1). Conversely, ohmefentanyl *trans*-(*2S*,*3R*,*4S*) exhibits a different spectrum with the peak at 79 cm^−1^ because of π-π stacking interaction from phenyl ring in R1, which is absent in *cis*-(*2R*,*3R*,*4S*) and *cis*-(*2S*,*3R*,*4R*).

**Figure 6:**
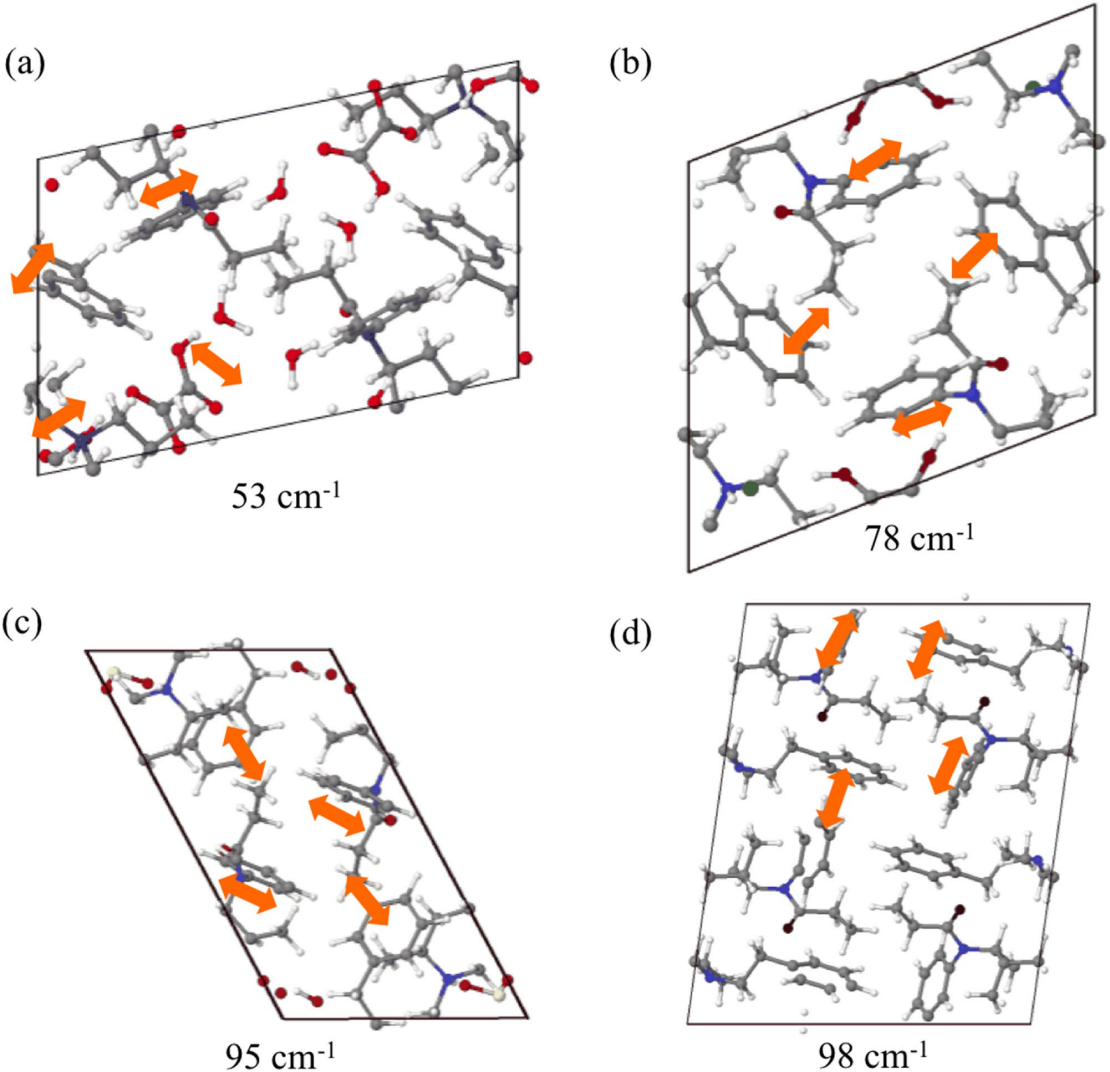
Normal modes of (**a**) FOPFIZ in 53 cm^−1^ along with a-axis, (**b**) CEWDEN10 in 78 cm^−1^ along with a-axis, (**c**) JINPAX in 95 cm^−1^ along with c-axis, and (**d**) VEYCIL in 98 cm^−1^ along with b-axis. Strongest peaks in THz range are shown. Carbon, hydrogen, nitrogen, and oxygen atoms are depicted as grey, white, blue, and red spheres, respectively.

Comparison between FOPFIZ (*trans*) and CEWDEN10 (*cis*) is presented in Fig. 5b. Both of them have several small peaks with one strong peak at 53 or 78 cm^−1^. Because of the solvent molecules and/or counterion in FOPFIZ and CEWDEN10, small and medium peaks are present to reflect the formation of multiple H-bonds. The strongest peak of FOPFIZ at 53 cm^−1^ shows the T-shaped phenyl ring interactions (see Fig. 6a). The strongest peak of CEWDEN10 at 78 cm^−1^ shows interactions between the dangling ethyl groups instead of phenyl rings (CH_3_…π interaction, Fig. 6b).

JINPAX (*2R*,*5S*) and VEYCIL (*2S*,*5S*) in Fig. 5c have similar situation that combine several small peaks with one or two strong peaks at 95 or 98 cm^−1^ (shown in Fig. 6c,d). THz spectra therefore distinguishes stereoisomers in these examples. JINPAX contains hydrogen sulfate that can form H-bond with nitrogen atom in piperidine ring. CH…π and parallel/T-shaped phenyl ring interactions determines the appearance of medium and strong peaks on both JINPAX and VEYCIL spectra.

All the other THz spectra of fentanyl analogs FBPIPA, BEGZUJ, IFIGIN, ZIZPON, and XALTAF can be found in Fig. S3. Strongest peaks above 75 cm^−1^ are based upon parallel π-π stacking, and T-shaped CH…π, CH_3_…π interactions. Note that there is no significant H-bond interaction in these analogs. Medium and small peaks below 75 cm^−1^ arise from long-distance, displaced stacking π-π and T-shaped interactions or from solvent molecules and counterions.

## Conclusions

Spectra of fentanyl and its analogs in THz range have specific characteristics. First, the generic picture of THz spectra of fentanyl and its analogs is that between 75–100 cm^−1^, there are 2–4 strong peaks from significant π-π interactions and/or H-bonds. Second, THz range between 20–75 cm^−1^ consists of small to medium peaks because of non-ideal π-π interactions, H-bond, and dispersion interaction. Third, the existence of π-π interactions or H-bonds that belong to solvent molecules or counterions leads to medium or strong peaks between 20–75 cm^−1^. Compared with compounds that are in white powder form (see Fig. S1), THz spectra of fentanyl and its analogs have the preference of peaks at certain THz range. The three characteristics may help to apply ML to predict unknown street drugs in the future and enable the detector design for synthetic opioids.

## Supplementary Information

Supplementary Information 1.
