# Supplementary material for: Accurate prediction of terahertz spectra of molecular crystals of fentanyl and its analogs
Source: Sci Rep. 2021 Feb 18;11:4062. doi: 10.1038/s41598-021-83536-y (PMC7892882; doi:10.1038/s41598-021-83536-y)
Supplement: Supplementary file 1 — Supplementary Information 1. [file 41598_2021_83536_MOESM1_ESM.pdf]

Supporting Information

Accurate prediction of terahertz spectra of molecular crystals of fentanyl and its analogs.

Chun-Hung Wang[1], Anthony C. Terracciano[2,3] , Artem E. Masunov*[1,4,5], Mengyu Xu[3,5], and Subith S. Vasu[2,3]

[1]NanoScience Technology Center, University of Central Florida, 12424 Research Parkway, Orlando FL, 32826, USA

[2]Department of Mechanical and Aerospace Engineering, University of Central Florida, 4000 Central Florida Blvd, Orlando FL, 32816, USA

[3]Center for Advanced Turbomachinery and Energy Research, University of Central Florida, 4000 Central Florida Blvd, Orlando FL, 32816, USA

[4]South Ural State University, Lenin pr. 76, Chelyabinsk 454080, Russia

[5]National Research Nuclear University MEPhI, Kashirskoye shosse 31, Moscow, 115409, Russia

[6]Department of Statistics, University of Central Florida, 4000 Central Florida Blvd, Orlando FL, 32816, USA

*Corresponding: amasunov@ucf.edu

Keywords: density functional theory, fentanyl, opioid, PBEh-3c, phonon, THz spectra

Validation of the accuracy of selected method and basis set

We first considered the benchmark study of pyridinium-containing molecular crystals by experimental and various computational methods.[1] Some of these methods and basis sets were tested in this study. As the paper[1] was published in 2016, we searched for any new developments in this field, and tested alternative basis sets. For simulations of Me4CP•Cl crystal, we had chosen HF-3c/MINIX[2], B3LYP/def2-mSVP[3-6], PBE0-D3/def2-SVP[7-11], PBE0-D3/def2-mSVP, PBE0-D3/def2-SVP[12], and PBEh-3c/def2-mSVP[5,6] basis sets. For simulations of Me4CP•Cl•H_2O crystal, we had chosen HF-3c/MINIX, B3LYP/def2-mSVP, M06-2X/def2-mSVP[13], PBE0-D3/def2-mSVP, PBE0-D3/pob-DVZP[14], ωB97-X/def2-mSVP[15], and PBEh-3c/def2-mSVP basis sets. According to Ruggiero *et al*,[1] ωB97-X/6-31G(d,p) produced the best agreement with experimental spectra. However, this basis set requires longer simulation time. We found that PBEh-3c/def2-mSVP produces computationally affordable yet consistent results. All the peaks are systematically red-shifted within 5-17 cm^{-1}. PBEh-3c includes both dispersion energy and basis set superposition error correction and is expected to provide the best option for molecular crystals in this study. In the following, PBEh-3c/def2-mSVP is benchmarked against the experimental THz spectra.

Eight amino acids (*L*-form glycine, cysteine, serine, asparagine, glutamine, alanine, leucine, and arginine), 4 saccharides (sucrose, lactose, trehalose, and mannitol), and 3 organic compounds (haloperidol, aspirin, and aspartame), whose structures can be found in Cambridge Crystallographic Database[16], were chosen to validate PBEh-3c/def2-mSVP computational method. The reason of selecting these molecules is availability of their room temperature experimental THz spectra in RIKEN, National Institute of Information and Communications Technology (http://thzdb.org), and NIST databases (https://webbook.nist.gov/chemistry/thz-ir). Since fentanyl analogs include amide bonds, THz spectra of amino acids provide helpful information for further comparison. Saccharides and the 3 organic molecules are “white powder” that can serve as controls for future validation of the future portable THz detector. Tables S1 and S2 show unit cell parameters of experimental and optimized structures respectively. The change of unit cell volume reported in Table S2 shows the geometry optimization results in at most 3.0% difference. Fig. S1 provides the comparison between predicted THz spectra of glycine, leucine, sucrose, and aspirin with experimental data from RIKEN or NIST databases. All the experimental spectra are baseline corrected for comparison purposes. The predicted position of each peak matches well with experimental ones (notwithstanding systematic ~1-20 cm^{-1} red shifts). In broader view, the method and basis set are appropriate to predict THz spectra of molecular crystals and is therefore applied in our study fentanyl and its analogs.

Table S1. Experimental unit cell parameters of 8 amino acids, 4 saccharides, and 3 organic compounds

	a [Å]	b [Å]	c [Å]	α [°]	β [°]	γ [°]	Vol. [$Å^3$]
Glycine	5.09	6.27	5.38	90.00	113.19	90.00	158.17
Cysteine	9.44	5.22	11.34	90.00	109.00	90.00	528.47
Serine	5.61	8.59	9.35	90.00	90.00	90.00	450.58
Asparagine	5.06	6.70	8.05	90.00	91.71	90.00	273.06
Glutamine	5.10	7.76	15.99	90.00	90.00	90.00	633.42
Alanine	6.04	12.34	5.79	90.00	90.00	90.00	431.18
Leucine	9.56	5.30	14.52	90.00	94.20	90.00	733.97
Arginine	9.76	16.02	5.58	90.00	98.06	90.00	863.77
Sucrose	7.72	8.68	10.82	90.00	102.98	90.00	706.98
Lactose	4.93	13.27	10.78	90.00	91.55	90.00	705.29
Trehalose	6.80	11.64	18.58	90.00	90.00	90.00	1470.61
Mannitol	4.87	8.87	18.74	90.00	90.00	90.00	808.96
Haloperidol	7.82	9.01	27.21	90.00	90.30	90.00	1917.12
Aspirin	11.23	6.54	11.23	90.00	95.89	90.00	821.22
Aspartame	19.41	4.96	15.65	90.00	94.88	90.00	1501.66

Table S2. Optimized unit cell parameters of 8 amino acids, 4 saccharides, and 3 organic compounds

	a [Å]	b [Å]	c [Å]	α [°]	β [°]	γ [°]	Vol. [$Å^3$]	∆Vol. %
Glycine	5.15	6.24	5.40	90.00	111.53	90.00	161.17	1.89
Cysteine	9.44	5.26	11.28	90.00	107.65	90.00	532.83	0.82
Serine	5.72	8.50	9.50	90.00	90.00	90.00	462.22	2.58
Asparagine	5.13	6.74	8.11	90.00	90.88	90.00	280.12	2.59
Glutamine	5.14	7.82	15.95	90.00	90.00	90.00	641.10	1.21
Alanine	6.10	12.14	5.85	90.00	90.00	90.00	433.16	0.46
Leucine	9.55	5.34	14.53	90.00	95.34	90.00	738.13	0.57
Arginine	9.78	16.17	5.50	90.00	97.16	90.00	863.21	-0.07
Sucrose	7.73	8.71	11.07	90.00	102.65	90.00	726.96	2.83
Lactose	4.96	13.40	10.88	90.00	91.57	90.00	723.63	2.60
Trehalose	6.77	11.76	18.60	90.00	90.00	90.00	1481.63	0.75
Mannitol	4.94	8.78	19.11	90.00	90.00	90.00	828.34	2.40
Haloperidol	7.80	9.01	26.65	90.00	89.32	90.00	1871.84	-2.36
Aspirin	11.20	6.57	11.60	90.00	99.12	90.00	842.80	2.63
Aspartame	19.26	5.06	15.42	90.00	93.87	90.00	1498.97	-0.18

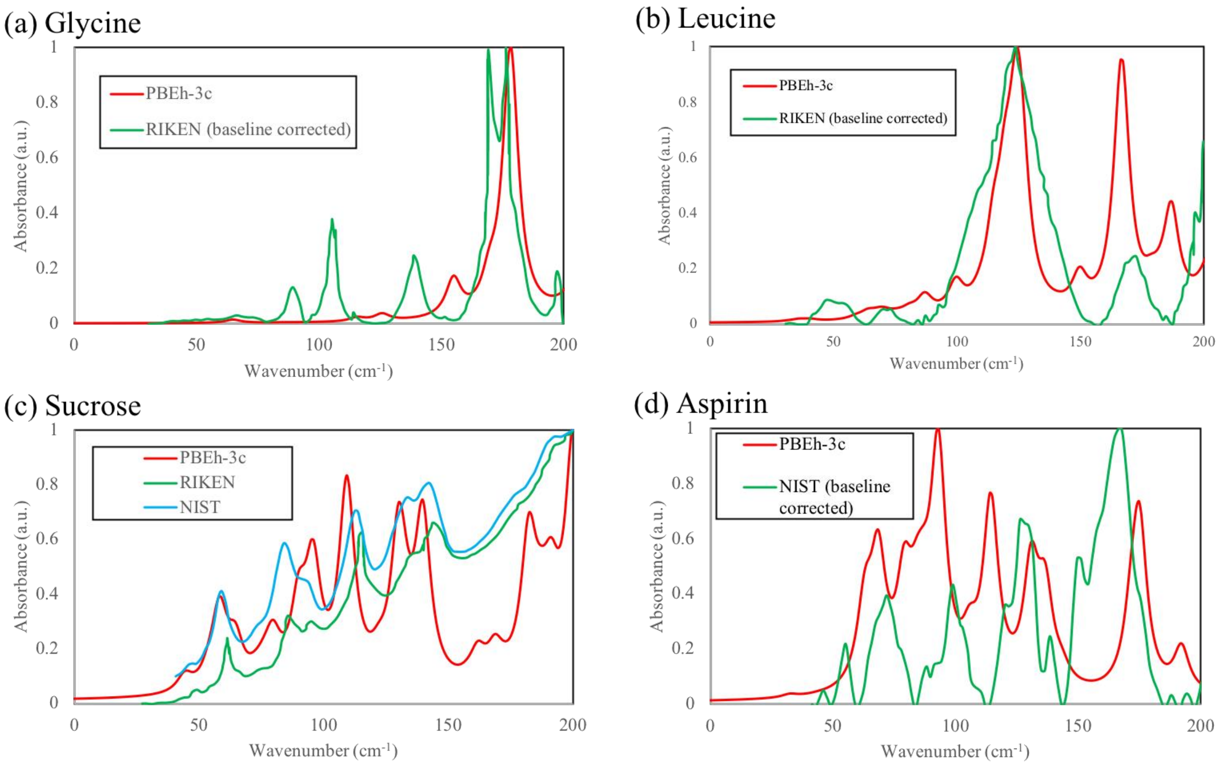

Figure S1. THz spectra of (a) glycine, (b) leucine, (c) sucrose, and (d) aspirin by computed PBEh-3c/def2-mSVP method or experimental results from RIKEN or NIST database.

Table S3. Experimental unit cell parameters of fentanyl and its selected analogs

	**a [Å]**	**b [Å]**	**c [Å]**	**α [°]**	**β [°]**	**γ [°]**	**Vol. [Å^3]**
Fentanyl	5.69	25.19	13.86	90.00	104.20	90.00	1926.47
3-Methylfentanyl	10.79	8.13	16.05	90.00	106.61	90.00	1348.12
BIYTAF	12.10	11.56	15.01	90.00	101.14	90.00	2061.07
TACDOS	9.30	9.72	14.42	78.62	75.81	74.62	1206.84
TACDUY	9.38	14.83	17.42	85.66	78.99	76.61	2313.31
FBPIPA	9.86	17.22	6.58	93.69	103.85	100.91	1058.30
BEGZUJ	17.02	9.75	8.73	119.08	95.40	93.64	1250.96
IFIGIN	10.88	22.47	11.53	90.00	92.21	90.00	2816.78
ZIZPON	11.05	12.11	20.51	75.97	78.97	94.80	2587.70
XALTAF	11.75	29.51	8.97	90.00	90.00	90.00	3110.20
R-30490	15.97	9.40	15.14	90.40	91.32	93.18	2266.24
Sufentanil	9.05	9.29	12.98	79.84	81.29	82.46	1056.21
Alfentanil	13.39	12.41	15.06	90.00	103.11	90.00	2438.42
Thiofentanil	8.56	10.30	13.89	99.25	86.64	107.68	1151.39
FOPFIZ (*trans*)	8.24	10.55	17.11	107.72	95.73	90.63	1406.86
CEWDEN10 (*cis*)	7.01	13.19	14.31	111.27	99.15	93.52	1206.29
JINPAX (*2R*,*5S*)	15.80	9.94	8.68	90.91	74.15	109.25	1233.64
VEYCIL (*2R*,*5R*)	15.29	7.36	19.46	90.00	97.71	90.00	2169.07
Ohmefentanyl							
cis-(*2R*,*3R*,*4S*)	7.58	11.73	23.21	90.00	90.00	90.00	2065.20
trans-(*2S*,*3R*,*4S*)	8.40	12.71	20.06	90.00	90.00	90.00	2141.94
cis-(*2S*,*3R*,*4R*)	7.61	11.74	23.23	90.00	90.00	90.00	2075.29

Table S4. Optimized unit cell parameters of fentanyl and its selected analogs

	**a [Å]**	**b [Å]**	**c [Å]**	**α [°]**	**β [°]**	**γ [°]**	**Vol. [Å^3]**	**ΔVol. %**
Fentanyl	5.54	27.71	12.98	90.00	102.55	90.00	1946.35	1.03
3-Methylfentanyl	10.77	8.14	16.07	90.00	108.05	90.00	1339.79	-0.62
BIYTAF	12.00	11.36	14.91	90.00	100.41	90.00	2005.64	-2.69
TACDOS	9.47	9.29	14.98	78.31	75.91	74.23	1217.26	0.86
TACDUY	9.44	14.81	17.41	85.74	78.23	77.00	2321.17	0.34
FBPIPA	9.82	17.09	6.54	93.29	103.57	101.72	1037.55	-1.96
BEGZUJ	17.09	9.56	8.65	118.30	93.06	93.98	1235.13	-1.27
IFIGIN	10.86	22.46	11.34	90.00	95.80	90.00	2750.50	-2.35
ZIZPON	11.13	12.09	20.29	76.67	76.03	93.75	2557.50	-1.17
XALTAF	11.85	28.57	8.97	90.00	90.00	90.00	3035.78	-2.39
R-30490	15.41	9.48	15.05	89.03	90.92	94.16	2191.25	-3.31
Sufentanil	9.07	9.12	12.49	82.78	82.35	82.58	1009.12	-4.46
Alfentanil	13.29	12.29	15.20	90.00	101.00	90.00	2437.91	-0.02
Thiofentanil	8.68	10.14	13.61	102.61	81.96	109.13	1100.82	-4.39
FOPFIZ (*trans*)	9.66	10.46	16.00	100.74	92.62	101.22	1551.43	10.28
CEWDEN10 (*cis*)	7.14	13.09	14.22	109.77	101.72	94.09	1210.83	0.38
JINPAX (*2R*,*5S*)	19.05	10.62	8.63	114.60	59.82	125.06	1215.00	-1.51
VEYCIL (*2R*,*5R*)	15.23	7.38	18.75	90.00	98.03	90.00	2088.00	-3.74
Ohmefentanyl								
cis-(*2R*,*3R*,*4S*)	7.41	11.42	23.36	90.00	90.00	90.00	1976.95	-4.27
trans-(*2S*,*3R*,*4S*)	8.15	12.95	19.59	90.00	90.00	90.00	2066.02	-3.54
cis-(*2S*,*3R*,*4R*)	7.41	11.42	23.35	90.00	90.00	90.00	1976.89	-4.74

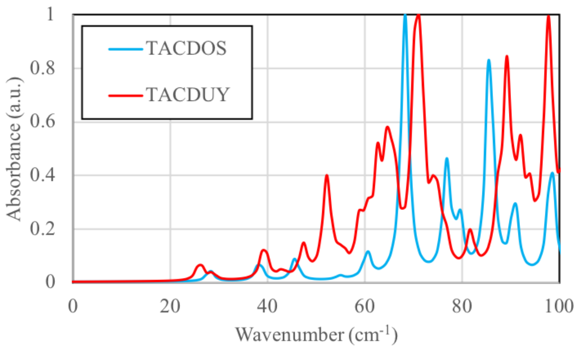

Figure S2. Predicted THz spectra of TACDOS (without solvents water and methanol) and TACDUY (with solvents water and methanol).

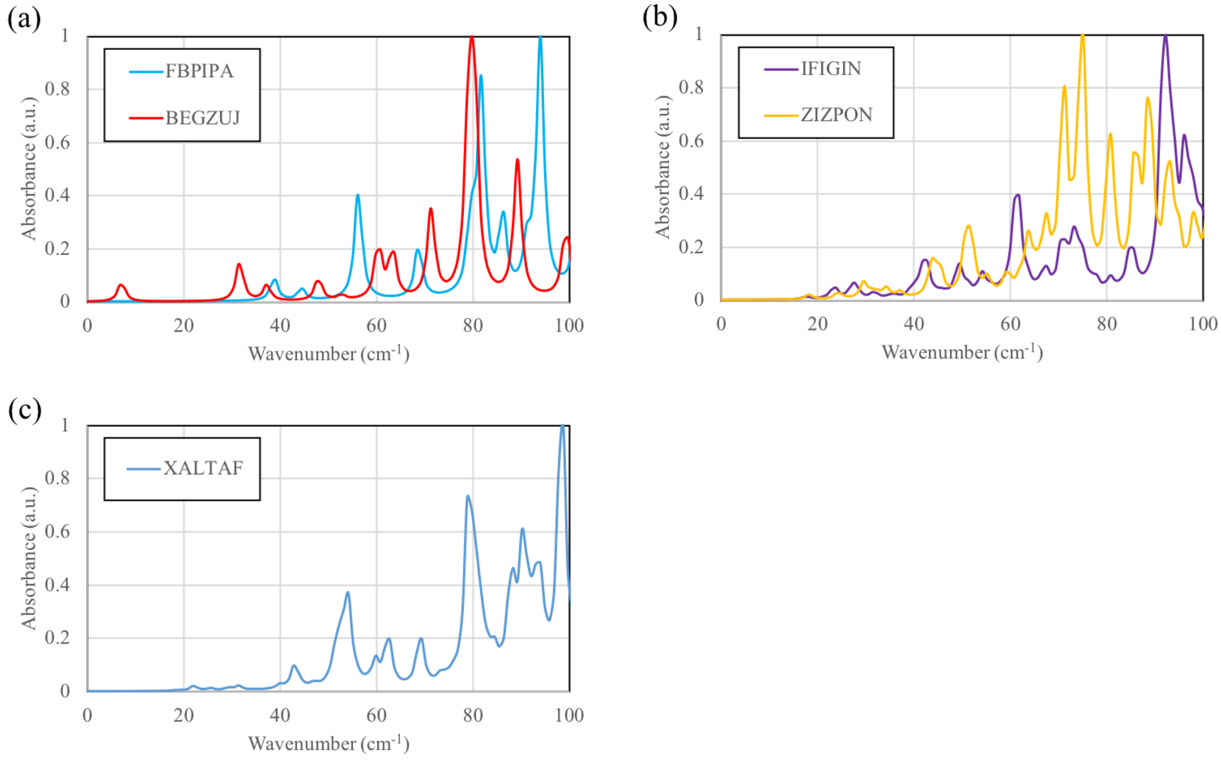

Figure S3. Predicted THz spectra of (a) FBPIPA and BEGZUJ, (b) IFIGIN and ZIZPON, and (c) XALTAF.

References

1 Ruggiero, M. T., Gooch, J., Zubieta, J. & Korter, T. M. Evaluation of Range-Corrected Density Functionals for the Simulation of Pyridinium-Containing Molecular Crystals. *Journal of Physical Chemistry A* **120**, 939-947 (2016).

2 Sure, R. & Grimme, S. Corrected Small Basis Set Hartree-Fock Method for Large Systems. *Journal of Computational Chemistry* **34**, 1672-1685 (2013).

3 Lee, C., Yang, W. & Parr, R. G. Development of the Colle-Salvetti correlation-energy formula into a functional of the electron density. *Physical Review B* **37**, 785-789 (1988).

4 Sure, R., Brandenburg, J. G. & Grimme, S. Small Atomic Orbital Basis Set First-Principles Quantum Chemical Methods for Larger Molecular and Periodic Systems: A Critical Analysis of Error Sources. *ChemistryOpen* **5**, 94-109 (2016).

5 Schäfer, A., Horn, H. & Alhrichs, R. Fully optimized contracted Gaussian basis sets for atoms Li to Kr. *Journal of Chemical Physics* **97**, 2571-2577 (1992).

6 Grimme, S., Brandenburg, J. G., Bannwarth, C. & Hansen, A. Consistent structures and interactions by density functional theory with small atomic orbital basis sets. *Journal of Chemical Physics* **143**, 054107 (2015).

7 Grimme, S., Antony, J., Ehrlich, S. & Krieg, H. A consistent and accurate ab initio parametrization of density functional dispersion correction (DFT-D) for the 94 elements H-Pu. *Journal of Chemical Physics* **132**, 154104 (2010).

8 Grimme, S., Ehrlich, S. & Goerigk, L. Effect of the damping function in dispersion corrected density functional theory. *Journal of Computational Chemistry* **32**, 1456-1465 (2011).

9 Grimme, S., Hansen, A., Brandenburg, J. G. & Bannwarth, C. Dispersion-Corrected Mean-Field Electronic Structure Methods. *Chemical Reivews* **116**, 5105-5154 (2016).

10 Perdew, J. P., Ernzerhof, M. & Burke, K. Rationale for mixing exact exchange with density functional approximations. *Journal of Chemical Physics* **105**, 9982-9985 (1996).

11 Adamo, C. & Barone, V. Toward reliable density functional methods without adjustable parameters: The PBE0 model. *Journal of Chemical Physics* **110**, 6158-6170 (1999).

12 Weigend, F. & Ahlrichs, R. Balanced basis sets of split valence, triple zeta valence and quadruple zeta valence quality for H to Rn: Design and assessment of accuracy. *Physical Chemistry Chemical Physics* **7**, 3297-3305 (2005).

13 Zhao, Y. & Truhlar, D. G. The M06 suite of density functionals for main group thermochemistry, thermochemical kinetics, noncovalent interactions, excited states, and transition elements: two new functionals and systematic testing of four M06-class functionals and 12 other functionals. *Theretical Chemistry Accounts* **120**, 215-241 (2008).

14 Peintinger, M. F., Oliveira, D. V. & Bredow, T. Consistent Gaussian basis sets of triple-zeta valence with polarization quality for solid-state calculations. *Journal of Computational Chemistry* **34**, 451-459 (2013).

15 Chai, J.-D. & Head-Gordon, M. Systematic optimization of long-range corrected hybrid density functionals. *Journal of Chemical Physics* **128**, 084106 (2008).

16 Groom, C., R., Bruno, I. J., Lightfoot, M. P. & Ward, S. C. The Cambridge Structural Database. *Acta Crystallographica Section B* **72**, 171-179 (2016).
